# The metabotropic glutamate receptor subtype 1 regulates development and maintenance of lemniscal synaptic connectivity in the somatosensory thalamus

**DOI:** 10.1371/journal.pone.0226820

**Published:** 2019-12-27

**Authors:** Madoka Narushima, Yuki Yagasaki, Yuichi Takeuchi, Atsu Aiba, Mariko Miyata

**Affiliations:** 1 Division of Neurophysiology, Department of Physiology, School of Medicine, Tokyo Women's Medical University, Shinjuku-ku, Tokyo, Japan; 2 Laboratory of Animal Resources, Center for Disease Biology and Integrated Medicine, Graduate School of Medicine, The University of Tokyo, Bunkyo-ku, Tokyo, Japan; Western University of Health Sciences, UNITED STATES

## Abstract

The metabotropic glutamate receptor subtype 1 (mGluR1) is a major subtype of group I mGluRs, which contributes to the development and plasticity of synapses in the brain. In the sensory thalamus, the thalamocortical neuron receives sensory afferents and massive feedback input from corticothalamic (CT) fibers. Notably, mGluR1 is more concentrated in CT synapses in the sensory thalamus. In the visual thalamus, mGluR1 maintains mature afferent synaptic connectivity. However, it is unknown whether mGluR1 contributes to strengthening of immature synapses or weakening of excess synapses during development and whether mGluR1 at CT synapses heterosynaptically regulates the development or refinement of afferent synapses. Here we investigated the effects of knocking out the gene encoding mGluR1 or pharmacologically blocking cortical activity on the development and maintenance of lemniscal synapses, i.e., the somatosensory afferent synapses, in the ventral posteromedial somatosensory thalamus. mGluR1-knockout (KO) mice exhibited delayed developmental strengthening as well as incomplete elimination and remodeling after maturation of lemniscal synapses. Similar to the phenotypes exhibited by mGluR1-KO mice, pharmacological blockade of somatosensory cortical activity from P12 or P21 for 1 week in wild-type mice perturbed elimination or maintenance of lemniscal synapses, respectively. The same manipulation in mGluR1-KO mice failed to induce additional abnormalities in lemniscal synaptic connectivity. These results suggest that activation of mGluR1, driven by CT input, regulates multiple stages of the development of lemniscal synapses, including strengthening, refinement, and maintenance in the somatosensory thalamus.

## Introduction

The development of synaptic connectivity involves initial formation, strengthening, and maintenance of synapses or weakening, pruning, and repression of the formation of excess synapses. In the sensory areas of the brain in particular, spontaneous and sensory experience-dependent activities control the development and refinement of precise synaptic connectivity [1–5]. Such mechanisms are defined in the afferent synapses of the sensory thalamus, which are the relay center for sensory information [6–8]. Further, increased concentrations of intracellular Ca^2+^ [9], expression of activity-related molecules [10], and activation of intracellular signals [11, 12] in postsynaptic thalamocortical (TC) neurons contribute to the development and refinement of thalamic afferent synapses. Group I metabotropic glutamate receptors (mGluRs), which include mGluR subtype 1 (mGluR1) and subtype 5, are candidates for studies aimed at identifying the mechanism underlying activity-dependent development of synapses because these receptors transduce neuronal transmissions to intracellular signaling cascades through G_q/11_ or homer-1, which trigger various downstream signal transduction cascades that mediate multiple forms of plasticity of synapses [13–16].

In the visual thalamus, the experience-dependent maintenance of mature afferent synapses formed by retinal ganglion cell axons, but not their formation or elimination, depends on the activation of mGluR1 in TC neurons [17]. Moreover, failure to maintain synapses in mGluR1-knockout (KO) mice results in weakening of their synaptic strength and recruitment of newly formed synapses. Recruitment of newly formed synapses may represent homeostatic plasticity in response to weakening of synaptic strength because the total amplitude of retinogeniculate synaptic responses remains unchanged. Therefore, the essential role of mGluR1 in the thalamic afferent synapses may involve maintenance of existing synapses or pruning of excess synapses as well as regulation of synaptic strength. Further, mGluR1 is expressed in the sensory thalamus, particularly at the postsynaptic site of corticothalamic (CT) feedback synapses [17, 18], which serve as a source of feedback excitatory input to TC neurons. Moreover, CT synaptic input maintains mature retinogeniculate synaptic connectivity [19], consistent with data acquired using mGluR1-KO [17]. Therefore, CT input activates mGluR1, and this activation may regulate developmental strengthening of synapses and neural connectivity.

To identify multiple mGluR1 functions during the development of synapses, we focused on lemniscal fiber–TC neuron synapses in the ventral posteromedial nucleus (VPm), which serves as a thalamic relay center for somatosensation in which mGluR1 is expressed earlier than dLGN [17]. Elimination of surplus afferent synapses during development or remodeling after maturation, similar to that observed in dLGN, occurs in VPm [7, 20–22]. We found that mGuR1 was highly expressed in VPm at birth. In mGluR1-KO mice, lemniscal fiber–TC neuron synaptic connectivity was aberrant after maturation as well as at the initial strengthening and developmental elimination phases. In the elimination and the maintenance phases, thalamic mGluR1 was probably activated by input from the primary somatosensory (S1) cortex because inhibition of neuronal activity in the S1 cortex induced synaptic remodeling, whereas the effect was occluded with mGluR1 knockout. We, therefore, conclude that mGluR1 activity, possibly driven by input from CT, regulates the multiple phases of development of synapses required for constructing and maintaining the fine neuronal circuit connectivity.

## Materials and methods

### Animals

All experiments were approved by the Animal Care and Use Committee of Tokyo Women’s Medical University and performed according to institutional guidelines. P0–P69 wild-type (WT) (C57BL/6) and mGluR1-KO mice were used. We used transgenic mice that specifically express mGluR1β in cerebellar Purkinje cells (PCs) but not in other brain regions [17], including VPm (mGluR1β-rescue mice) [23].

### Slice preparations and whole-cell recordings

Mice were decapitated under isoflurane anesthesia. Sagittal brain slices, including VPm (250–300 μm thick), were prepared from C57BL/6 or mGluR1-KO mice in ice-cold cutting solution, containing 234 mM sucrose, 2.5 mM KCl, 1.25 mM NaH_2_PO_4_, 10 mM MgCl_2_, 0.5 mM CaCl_2_, 25 mM NaHCO_3_, 0.5 mM myoinositol, and 11 mM glucose, bubbled with 95% O_2_ and 5% CO_2_. For mice older than P60, a modified cutting solution, containing 130 mM K-gluconate, 15 mM KCl, 0.05 mM EGTA, 20 mM HEPES, 25 mM glucose, and 2.5 μM 3-((*R*)-2-carboxypiperazin-4-yl)-propyl-1-phosphonic acid [(R)-CPP] (pH 7.4, adjusted with NaOH), was used. The slices were recovered in artificial cerebrospinal fluid, containing 125 mM NaCl, 2.5 mM KCl, 1.25 mM NaH_2_PO_4_, 1 mM MgSO_4_, 2 mM CaCl_2_, 26 mM NaHCO_3_, and 20 mM glucose, equilibrated with 95% O_2_ and 5% CO_2_ at 32°C for 30 min and then kept at room temperature.

For whole-cell recording, a patch pipette (2.5–4 MΩ) was filled with intracellular solution, containing 120 mM CsMeSO_3_, 10 mM HEPES, 1 mM EGTA, 2 mM MgCl_2_, 0.1 mM CaCl_2_, 20 mM NaCl, 5 mM QX-314, 2 mM ATP-Na_2_, 0.5 mM GTP-Na, and 0.5% biocytin (pH 7.4, adjusted with CsOH), 290 mOsm. The perfusate contained 10 μM (-)-bicuculline methochloride, 1 μM CGP55845, and 1 μM strychnine (Tocris, UK). Recordings of neurons in VPm were obtained using the infrared–differential interference contrast view of an upright microscope (BX51WI; Olympus, Japan or AXIO Examiner A1; Carl Zeiss, Germany) equipped with an IR-CCD camera system (IR-1000; DAGE-MTI, USA). Membrane currents were recorded using an EPC10 amplifier (HEKA, Germany).

### Recordings and evaluation of lemniscal excitatory postsynaptic currents (EPSCs)

A concentric bipolar electrode was placed on the medial lemniscal fiber bundle; two successive square pluses with a 100-ms interpulse interval were delivered at 0.1 Hz (100 μs, typically 10–400 μA). Lemniscal EPSCs are characterized by paired-pulse depression of responses to the second stimuli and all-or-none or stepwise increments with distinct thresholds in response to increasing stimulus intensity. To determine the number of lemniscal inputs for each thalamic neuron, lemniscal EPSCs were evoked at −70 and +40 mV (AMPAR- and NMDAR-medicated EPSCs, respectively) from the same neuron over a wide range of stimulus intensities [21, 22]. The single-fiber (SF) EPSC amplitude of each lemniscal fiber was defined as the difference in the amplitude of two successive EPSC steps induced by subthreshold and suprathreshold stimuli for that axon. The SF fraction was calculated as the ratio of the SF EPSC amplitude to the maximum EPSC amplitude at a holding potential of −70 mV for each cell. The SF paired-pulse ratio (PPR) was calculated by the amplitude of two successive AMPAR-mediated EPSCs with interpulse interval of 100 ms. The step number of lemniscal EPSCs was counted with EPSCs evoked at −70 mV.

### Perturbation of cortical activity by muscimol

To continuously apply muscimol in vivo, we used EVAFLEX (EV40W, DuPont-Mitsui Polychemicals, Japan), the commercial counterpart of ELVAX. Pieces of EVAFLEX (1 mm × 1 mm × 0.2 mm) were prepared [22]; each piece contained the vehicle or 100 mM of muscimol. To implant the pieces of EVAFLEX, mice were anesthetized using isoflurane; a craniotomy (1.5 mm×1.5 mm) was performed above the primary somatosensory cortex at P12 or P21 in WT and mGluR1-KO mice. After removal of dura using a needle, a piece of EVAFLEX was placed onto the surface of the brain. The craniotomy was sealed with Kwik-Cast (World Precision Instruments, USA), covered with dental cement, and sutured.

### Immunohistochemistry

Mice were anesthetized using pentobarbital (50 mg/kg intraperitoneally) and perfused with freshly prepared 4% paraformaldehyde and 0.2% picric acid in 0.1 M phosphate buffer (pH 7.4). After perfusion, the brain was removed and fixed using the same solution overnight and permeated with 10%–30% sucrose in 0.1 M phosphate buffer (pH 7.5). The samples were frozen in O.C.T. compound (Sakura Fine Technical, Japan) and stored at −80°C. The frozen samples were divided into 20-μm-thick sections using a cryostat (Leica CM1850; Leica Microsystems, Germany). The sections were incubated with 10% normal donkey serum for preventing nonspecific reactions and then incubated with an antibody against mGluR1α (1:500 diluted; mGluR1α-Rb-Af811, Frontier Institute, Japan). Alexa Fluor 594-conjugated donkey anti–rabbit IgG (A21207, Thermo Fisher Scientific, USA) served as the secondary antibody. Sections were counterstained with the fluorescent Nissl stain NeuroTrace 435/455 (N-21479, Thermo Fisher Scientific, USA). Images of fluorescence were taken with a fluorescence microscope (Axio Scope A1; Carl Zeiss, Germany) equipped with a 2.5× objective lens and acquired at 2758 × 2208 pixels using ImageJ software. To quantitatively evaluate the expression of mGluR1, fluorescence intensities in VPm and dLGN were normalized against those in the external medullary lamina using ImageJ software. The statistical significance of the differences was determined using Dunnett’s test for comparisons with P0. Statistical tests were performed with JMP Pro 14 software (SAS Institute Inc., Cary, NC, USA); P < 0.05 was considered as a significant difference.

## Results

### Expression of mGluR1 in the somatosensory thalamus

**Fig 1:**
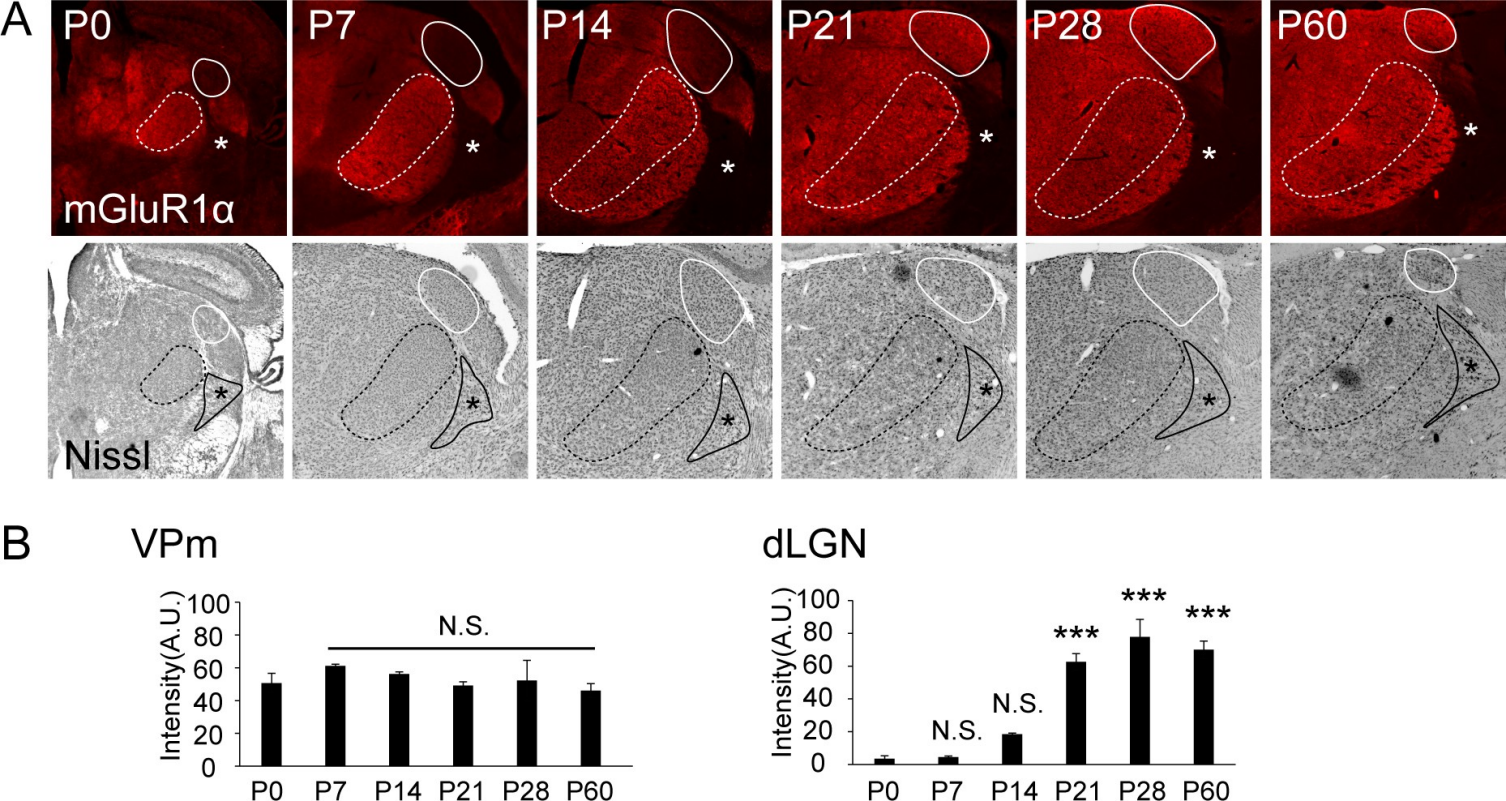
Developmental expression of mGluR1 in VPm and reticular nuclei. (A) mGluR1α immunohistochemical staining (top) and Nissl staining (bottom) in the thalamus of WT mice at P0, P7, P14, P21, P28, and P60. The white and black dotted lines, VPm: ventral posteromedial nucleus. The white lines, dLGN: dorsal lateral geniculate nucleus. The black lines and asterisks, RTN: reticular thalamic nucleus. Scale bar = 500 μm. (B) Developmental changes in the expression of mGluR1 in VPm (left) and dLGN (right). Fluorescence intensities in VPm and dLGN (N = 3 mice) were normalized against those in the external medullary lamina. A.U.: arbitrary unit. Data are presented as means ± SEM. *** represents p < 0.001 with Dunnett’s test for comparisons with P0. N.S.: not significant.

First, we analyzed the expression of mGluR1 in VPm during development. mGluR1 showed high expression in VPm from P10 to P30 [17]; however, its neonatal level of expression is unknown. Immunohistochemical staining revealed that mGluR1α was expressed in VPm at birth and during development to adulthood (Fig 1A). Conversely, in other thalamic nuclei, such as the reticular nucleus, which delivers inhibitory inputs to VPm, detectable expression of mGluR1α was not observed (Fig 1A). We compared the level of expression of mGluR1α during postnatal ages in VPm and dLGN (Fig 1B). Unlike dLGN, in which the expression of mGluR1α significantly increased from P21, the expression in VPm was stable among the sampled ages compared with that in VPm at P0. These results support the idea that mGluR1 contributes to the development of synapses in VPm.

### mGluR1 contributes to the strengthening of synapses during early development

**Fig 2:**
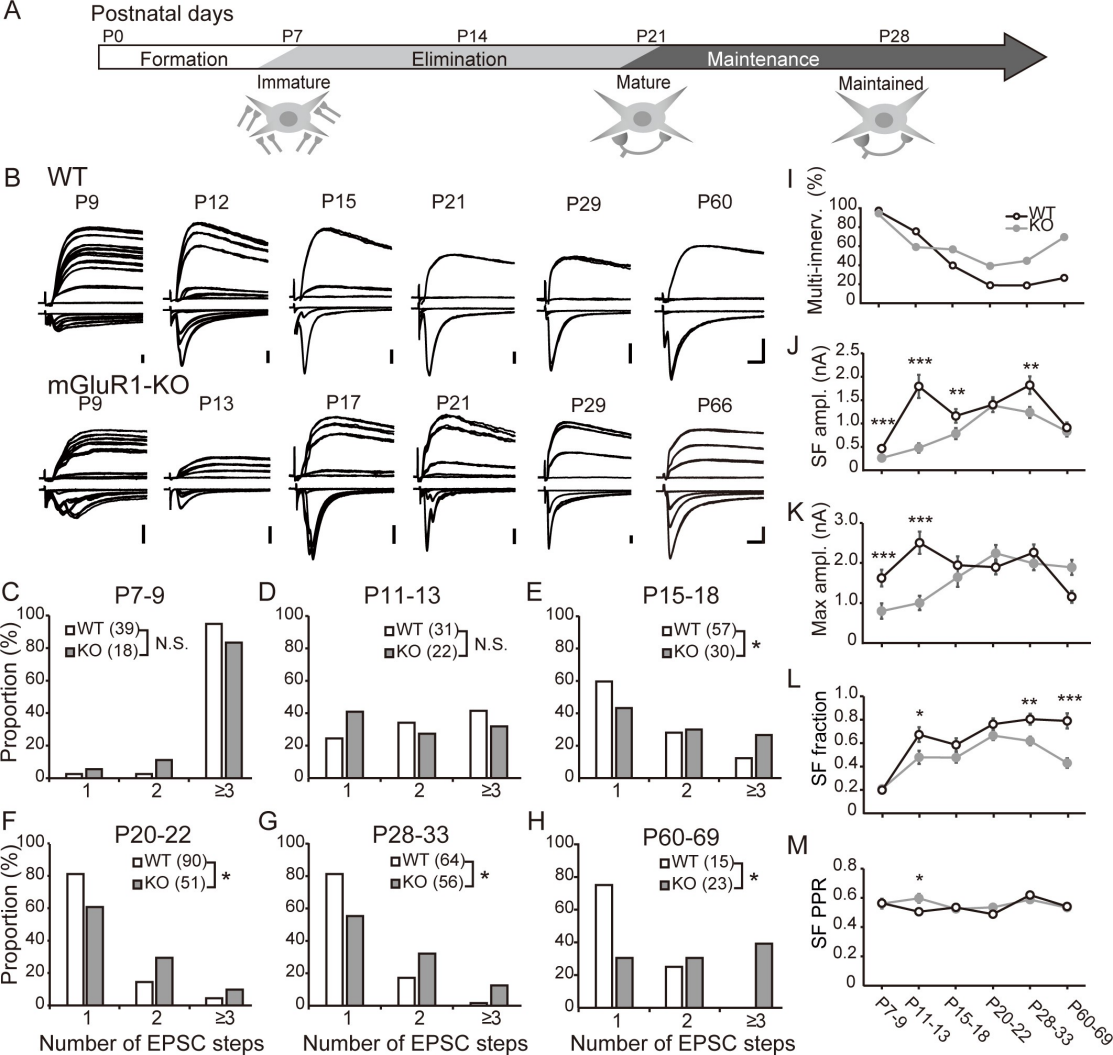
mGluR1 regulates multiple phases of development and maintains lemniscal synapses. (A) Developmental time course of the lemniscal synapse. After exuberant synapse formation, some synapses are strengthened; excess synapses are eliminated by P21. Most TC neurons in VPm are projected by a single lemniscal fiber after excess synapse elimination. This mono-innervation pattern is maintained in adults. (B) Representative traces of lemniscal EPSC recorded from TC neurons of WT (top) or mGluR1-KO mice (bottom) at −70 or +40 mV obtained at the indicated ages. Calibration bars = 0.5 nA and 5 ms. (C)–(H) Distribution of step number of lemniscal EPSCs in six age groups of WT (white) and mGluR1-KO mice (gray). Numbers of recorded TC neurons are indicated in brackets. *p < 0.05, chi-square test. (I) Developmental change in the proportion of TC neurons innervated by multiple lemniscal fibers in WT (open circles) and mGluR1-KO (filled circles) mice. (J)–(M) Developmental changes in the amplitude of AMPAR-mediated EPSC induced by SF stimulation (J), the maximum AMPAR-mediated EPSC amplitude (K), the SF fraction (L), and the PPR of SF AMPAR-mediated EPSCs (M). *p < 0.05, **p < 0.01, ***p < 0.001, Mann–Whitney test between strains of the same age group, respectively.

To investigate the function of mGluR1 in developing VPm, we analyzed electrophysiological properties of lemniscal EPSCs in WT and mGluR1-KO mice at various postnatal ages. We recorded AMPA receptor (AMPAR)- and NMDA receptor (NMDAR)-mediated EPSCs from TC neurons in response to stimulation of the lemniscal fiber. Because the release probability of transmitter from the presynaptic terminal of a lemniscal fiber is high, stimulation of the lemniscal fiber generates EPSCs in an all-or-none manner. Lemniscal EPSCs recorded from matured TC neuron typically exhibit a large, single step, suggesting that the TC neuron is innervated by a single lemniscal fiber (mono-innervation). Conversely, lemniscal EPSCs recorded from immature TC neurons show multiple stepwise increments in their amplitudes, suggesting that the TC neuron is innervated by multiple lemniscal fibers (multi-innervation, Fig 2A) [20, 21]. In WT mice at approximately P7, stepwise increments in EPSC amplitudes were frequently observed as a function of increased stimulus intensity for AMPAR- and NMDAR-mediated components (Fig 2B, top) as reported previously [20, 21]. The SF and maximum amplitude increased between P7–P9 and P11–P13 (Fig 2J and 2K, Tables 1 and 2). The AMPA/NMDA ratio increased between P7–P9 and P11–P13 and subsequently stabilized (Table 2). The SF PPR was mostly stable during development (Fig 2M).

**Table 1:** Development of AMPAR-mediated lemniscal synaptic currents in WT and mGluR1-KO mice.

	Strain	P7–P9	P11–P13	P15–P18	P20–P22	P28–P33	P60–P69
SF amplitude (nA)	WT	0.47 ± 0.06	1.79 ± 0.25	1.16 ± 0.15	1.40 ± 0.16	1.82 ± 0.19	0.92 ± 0.11
	KO	0.26 ± 0.07***	0.48 ± 0.10***	0.78 ± 0.12***	1.38 ± 0.14	1.24 ± 0.12**	0.83 ± 0.11
Max. amplitude (nA)	WT	1.62 ± 0.21	2.50 ± 0.28	1.94 ± 0.22	1.89 ± 0.18	2.26 ± 0.02	1.16 ± 0.15
	KO	0.80 ± 0.19***	1.00 ± 0.18***	1.64 ± 0.24	2.24 ± 0.21	1.98 ± 0.17	1.89 ± 0.19
SF fraction	WT	0.20 ± 0.02	0.64 ± 0.06	0.59 ± 0.06	0.76 ± 0.05	0.80 ± 0.05	0.79 ± 0.07**
	KO	0.21 ± 0.02	0.48 ± 0.06*	0.48 ± 0.04	0.66 ± 0.04	0.62 ± 0.04**	0.43 ± 0.04***
SF PPR	WT	0.56 ± 0.02	0.51 ± 0.03	0.53 ± 0.02	0.49 ± 0.01	0.61 ± 0.01	0.54 ± 0.02
	KO	0.56 ± 0.03	0.60 ± 0.03*	0.52 ± 0.02	0.54 ± 0.01*	0.59 ± 0.01	0.53 ± 0.02
Cell number	WT	21	21	21	30	33	15
	KO	18	22	30	44	51	23

Single-fiber amplitude, maximum amplitude, single-fiber fraction, and single-fiber paired-pulse ratio of AMPAR-mediated currents recorded at −70 mV and the number of recorded WT and mGluR1-KO (KO) cells at the indicated ages.

*p < 0.05

**p <0.01

***p < 0.001 (Mann–Whitney test) between strains of the same age group, respectively.

**Table 2:** Development of NMDAR-mediated lemniscal synaptic currents in WT and mGluR1-KO mice.

	Strain	P7–P9	P11–P13	P15–P18	P20–P22	P28–P33	P60–P69
SF amplitude (nA)	WT	0.49 ± 0.06	1.32 ± 0.17	1.15 ± 0.15	1.33 ± 0.12	1.99 ± 0.28	0.78 ± 0.11
	KO	0.30 ± 0.05***	0.39 ± 0.10***	0.78 ± 0.13*	1.63 ± 0.07	1.12 ± 0.14**	0.68 ± 0.09
Max. amplitude (nA)	WT	2.25 ± 0.21	1.98 ± 0.23	1.97 ± 0.22	1.55 ± 0.13	2.19 ± 0.26	1.00 ± 0.14
	KO	1.45 ± 0.25***	1.00 ± 0.21**	2.04 ± 0.22	2.05 ± 0.19	1.65 ± 0.16	1.49 ± 0.10**
SF-EPSC decay τ (ms)	WT	82.5 ± 2.9	63.0 ± 4.2	39.9 ± 2.1	43.1 ± 5.4	51.2 ± 3.4	51.3 ± 2.4
	KO	75.8 ± 3.1	54.2 ± 3.3	45.1 ± 2.6	55.2 ± 2.4*	46.6 ± 2.2	49.3 ± 2.3
SF AMPA/NMDA ratio	WT	0.86 ± 0.05	1.24 ± 0.08	1.10 ± 0.08	1.27 ± 0.10	1.17 ± 0.10	1.30 ± 0.11
	KO	0.66 ± 0.05**	1.00 ± 0.06*	1.29 ± 0.08*	1.21 ± 0.06	1.24 ± 0.06	1.47 ± 0.10
Cell number	WT	19	17	20	20	11	14
	-KO	17	16	21	26	27	20

Single-fiber amplitude, maximum amplitude, decay time constant of single-fiber responses of NMDA receptor-mediated currents recorded at +40 mV, AMPA/NMDA ratio of single-fiber responses, and number of recorded cells of WT and mGluR1-KO (KO) mice at the indicated ages.

*p < 0.05

**p <0.01

***p < 0.001, Mann–Whitney test, between strains of the same age group, respectively.

Stepwise increments in lemniscal EPSC amplitudes were also observed in mGluR1-KO mice at approximately P7 (Fig 2B, bottom). However, the lemniscal EPSC amplitude of each SF at P7–P9 (0.26 ± 0.07 nA, n = 18 cells) and the maximum amplitude of AMPAR-mediated EPSCs (0.80 ± 0.19 nA) of mGluR1-KO mice were significantly smaller than those of WT mice (p < 0.0001, Mann–Whitney test) (Fig 2J and 2K and Table 1). SF AMPAR- or NMDAR-mediated EPSC amplitudes remained smaller until P15–P18 (Fig 2J and Tables 1 and 2). The maximum EPSC amplitude showed a similar tendency (Fig 2K and Tables 1 and 2), and the AMPA/NMDA ratio was smaller in KO mice during P7–P9 or P11–P13 (Table 2). At approximately P20, the lemniscal EPSC amplitude of SF and the maximum EPSC of KO mice were similar to those of WT mice (Fig 2J and 2K and Tables 1 and 2), suggesting that mGluR1 contributes to strengthening of lemniscal synapses early during development. In mGluR1-KO mice, other mechanism(s) may compensate for strengthening of synapses in the subsequent stages of development to reach the WT level of synaptic strength.

### mGluR1 regulates the elimination and maintenance of lemniscal synapses

The reduction in the number of lemniscal EPSC steps serves as an index for quantifying the elimination and maintenance of synapses. In WT mice, the number of steps gradually decreased (Fig 2C–2H) with an increase in SF-EPSC amplitudes (Fig 2J, Tables 1 and 2), suggesting strengthening of required synapses and elimination of excess synapses. By P20, approximately 80% of TC neurons exhibited a single-step lemniscal EPSC, and this mono-innervation pattern was subsequently maintained (Fig 2F–2I). Thus, the SF fraction (i.e., the ratio of SF AMPAR-mediated EPSC amplitude to maximum AMPAR-mediated EPSC amplitude in a given cell) gradually increased (Fig 2L and Table 1). The maximum amplitude tended to decrease from P20–P22 to P60–P69 (Fig 2K, Tables 1 and 2). This tendency in adults can be explained by additional refinement of branches of afferent fibers similar to that in the visual thalamus [24].

During the early phase of development where the amplitude was smaller than that in WT mice, the number of EPSC steps in mGluR1-KO mice gradually decreased, similar to that observed in WT mice (Fig 2C–2F and 2I), and the SF fraction gradually increased (Fig 2L and Table 1) until P20–P22. These data indicate that developmental elimination of synapses gradually proceeded in mGluR1-KO mice. However, the shift in the distribution of step numbers from multiple to single innervations was slower (Fig 2E and 2F), and the proportion of mono-innervated TC neurons remained at approximately 60% in P20–P22 and P28–P33 mice (Fig 2F and 2G). Residual TC neurons were innervated by multiple lemniscal fibers even after P20, by which time developmental elimination in WT mice was completed (Fig 2I). Thus, the SF fraction of mGluR1-KO mice was lower than that of WT mice throughout development (Fig 2L and Table 1). These findings indicate that mGluR1-KO mice exhibited delayed and incomplete elimination of lemniscal synapses.

In the P60–P69 mGluR1-KO mice, the proportion of multi-innervated TC neurons increased to a similar extent as that in their P11–P13 counterparts (Fig 2H and 2I). In contrast to the reduction in the maximum amplitude in P60–P69 WT mice, the maximum amplitude in mGluR1-KO mice was comparable to that in their P28–33 counterpart and the SF amplitude continued to decrease (Fig 2J and 2K and Table 1). Thus, the SF fraction in mGluR1-KO mice was significantly smaller than that in WT mice (Fig 2L and Table 1), suggesting that in mGluR1-KO mice, mono-innervation of lemniscal fibers could not be maintained in combination with the increment in the number of input fibers innervating a single TC neuron. These results strongly suggest that mGluR1 contributes to the elimination of excess synapses as well as the maintenance of mature synaptic connectivity. Together, mGluR1 contributes to multiple stages of development, including strengthening, elimination, maturation, and maintenance of lemniscal synapses.

### Cortical activity regulates elimination and maintenance of lemniscal synapses

In the sensory thalamus, including VPm, mGluR1 is highly expressed in the distal portion of the dendrites of TC neurons where cortical fibers preferentially terminate and form abundant synapses [18, 25]. mGluR1 was expressed from P0 in VPm (Fig 1), although the expression around CT synapses dramatically increases between P7 and P9 [26]. Therefore, we hypothesized that mGluR1 is activated by cortical input from the S1 area to VPm, particularly during the elimination and maintenance of synapses. To provide evidence to support this possibility, we manipulated cortical activity through chronic treatment with muscimol, a GABA_A_ receptor agonist, to the S1 area of the cortex to reduce neuronal activity. EVAFLEX was used to continuously deliver muscimol.

**Fig 3:**
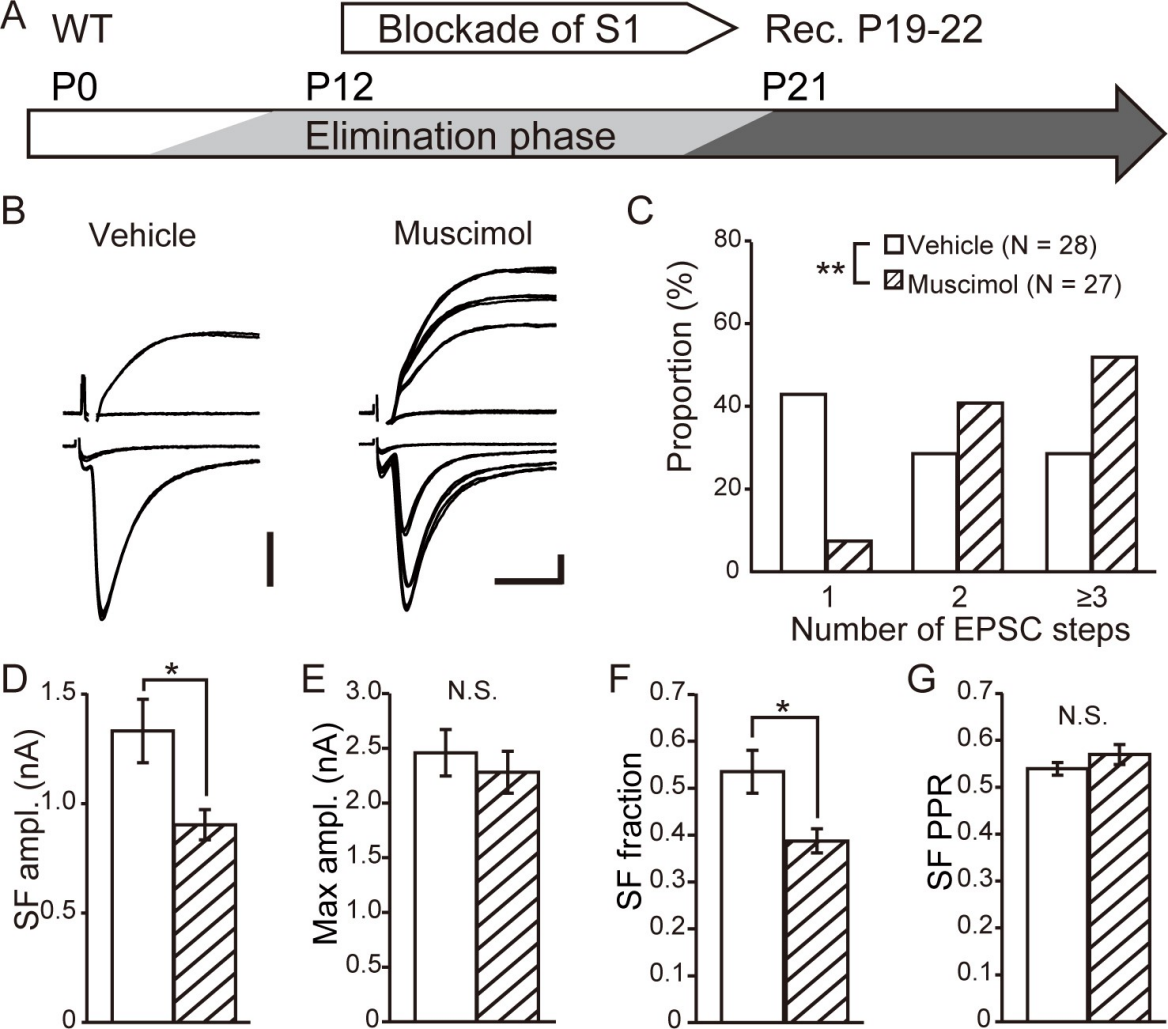
Developmental elimination of lemniscal synapses requires cortical activity. (A) Experimental schedule: muscimol was applied in the S1 area of the cortex from P12 in WT mice. Electrophysiological recordings were obtained from P19 to P22. (B) Representative traces of TC neurons obtained from vehicle-treated (left) or muscimol-treated (right) mice. Calibration bars = 0.5 nA and 5 ms. (C) Distribution of step numbers of lemniscal EPSCs after treatment with the vehicle (open) or muscimol (hatched). **p < 0.01, chi-square test. (D)–(G) Comparisons of the SF AMPAR-mediated EPSC amplitude (D), maximum AMPAR-mediated EPSC amplitude (E), SF fraction (F), and PPR of SF AMPAR-mediated EPSCs (G) between vehicle- (open) and muscimol-treated (hatched) mice. *p < 0.05, Mann–Whitney test. N.S., not significant.

First, we investigated the effects of inhibiting cortical activity upon elimination of lemniscal synapses. Muscimol treatment of WT mice commenced in P12 (elimination phase application), and electrophysiological recordings were obtained during P19–P22 when developmental elimination of synapses should have been nearly completed (Fig 3A). The proportion of TC neurons innervated by multiple lemniscal fibers was significantly higher in muscimol-treated mice than in vehicle-treated mice (p < 0.01, chi-square test) (Fig 3B and 3C). Similar to data acquired for mGluR1-KO mice, the SF-EPSC amplitude (vehicle, 1.33 ± 0.15 nA vs muscimol, 0.90 ± 0.07 nA; P = 0.030, Mann–Whitney test) (Fig 3D) or SF fraction (vehicle, 0.54 ± 0.05 vs muscimol, 0.39 ± 0.03; P = 0.030, Mann–Whitney test) (Fig 3F) was smaller although the maximum amplitude (vehicle, 2.46 ± 0.21 nA vs muscimol, 2.28 ± 0.19 nA; P = 0.87, Mann–Whitney test) and SF PPR were not significantly different (Fig 3E and 3G) in muscimol-treated mice. We interpreted these data to mean that a reduction in the cortical activity perturbed developmental elimination of lemniscal synapses.

**Fig 4:**
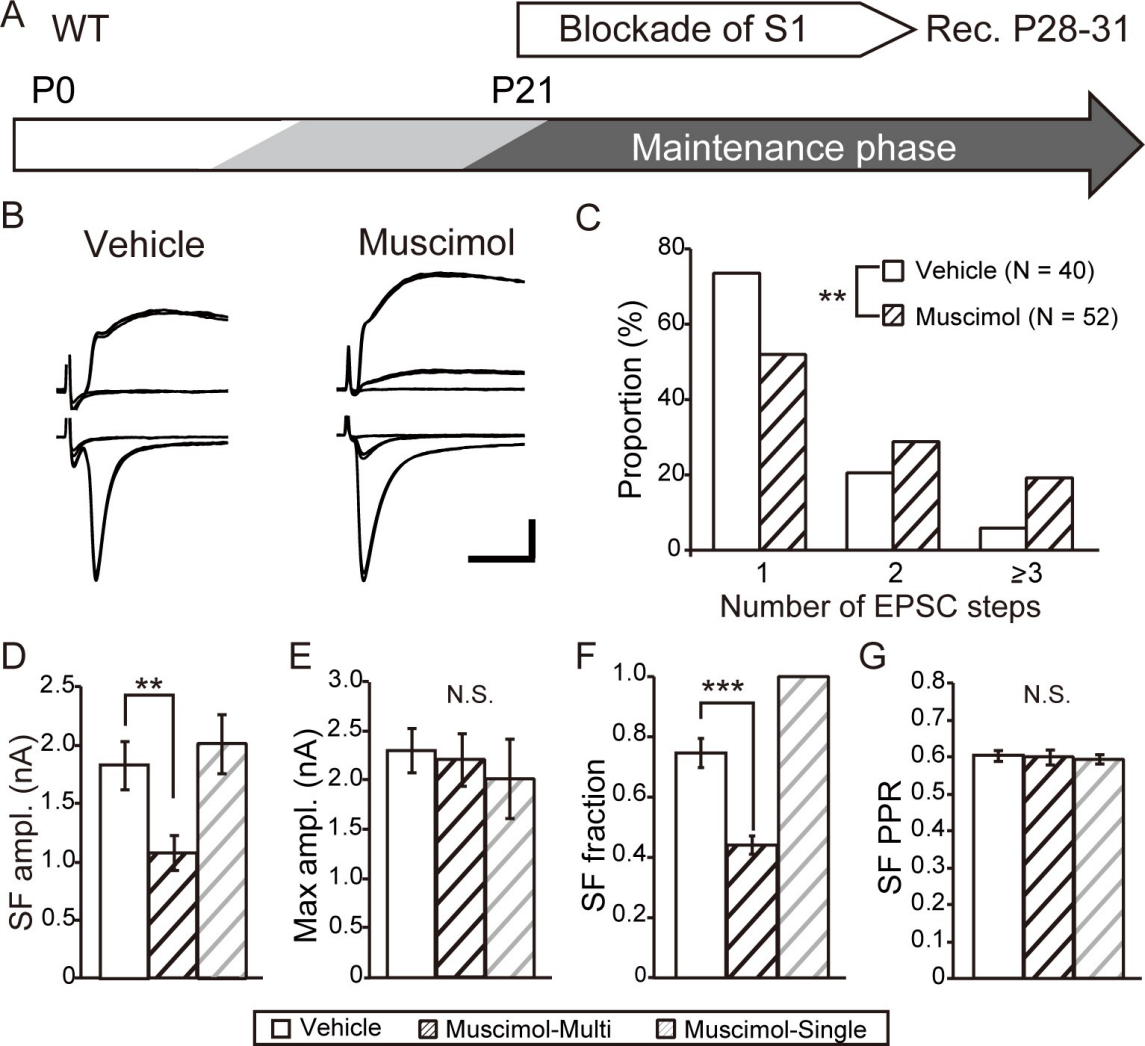
Maintenance of mature connectivity of lemniscal synapses requires cortical activity. (A) Experimental schedule: muscimol was applied in the S1 area of the cortex from P21 in WT mice. Electrophysiological recordings were obtained from P28 to P31. (B) Representative traces of TC neurons obtained from vehicle-treated (left) or muscimol-treated (right) mice. Calibration bars = 1 nA and 5 ms. (C) Distribution of step numbers of lemniscal EPSCs after vehicle (open) or muscimol (hatched) treatment. **p < 0.01, chi-square test. (D)–(G) Comparisons of the SF AMPAR-mediated EPSC amplitude (D), maximum AMPAR-mediated EPSC amplitude (E), SF fraction (F), and PPR of SF AMPAR-mediated EPSCs (G) among vehicle-treated mice (open) and multi-innervated TC neurons (hatched) and mono-innervated TC neurons (pale hatched) in muscimol-treated mice. **p < 0.01, ***p < 0.001, Mann–Whitney test. N.S., not significant.

Next, we applied muscimol on P21 mice after completion of developmental elimination of synapses (maintenance phase application) and then obtained electrophysiological recordings for lemniscal EPSCs from P28 to P31 of WT mice to determine whether cortical activity influenced the maintenance of lemniscal synapses (Fig 4A). Late application of muscimol induced an increase in the number of innervating lemniscal fibers per TC neuron (Fig 4B and 4C). TC neurons (48.1%) were projected by multiple lemniscal fibers after muscimol treatment, which was a significantly higher rate than that after vehicle treatment (26.3%, p < 0.01, chi-square test). For further analysis, we divided recordings from neurons in muscimol-treated mice depending on the number of projecting lemniscal fibers. The characteristics of lemniscal EPSCs in neurons innervated by a single lemniscal fiber in muscimol-treated mice were similar to those of lemniscal EPSCs in neurons of vehicle-treated mice (Fig 4D–4G). However, in neurons innervated by multiple lemniscal fibers, the SF AMPAR-mediated EPSC amplitude (vehicle, 1.83 ± 0.21 nA, n = 38 vs muscimol-multi, 1.08 ± 0.15 nA, n = 25; P = 0.002, Mann–Whitney test) (Fig 4D) and the SF fraction (vehicle, 0.86 ± 0.05 vs muscimol-multi, 0.40 ± 0.05; p < 0.0001, Mann–Whitney test) (Fig 4F) in muscimol-treated mice were significantly smaller than those in the vehicle-treated mice. These results suggest that cortical activity was required for the maintenance of precise lemniscal synaptic connectivity. The results of elimination phase application and that of maintenance phase application strongly support the conclusion that cortical activity regulates the connectivity of lemniscal synapse during developmental elimination and maintenance phase after maturation.

### Cortical regulation of lemniscal synaptic connectivity requires activation of mGluR1

**Fig 5:**
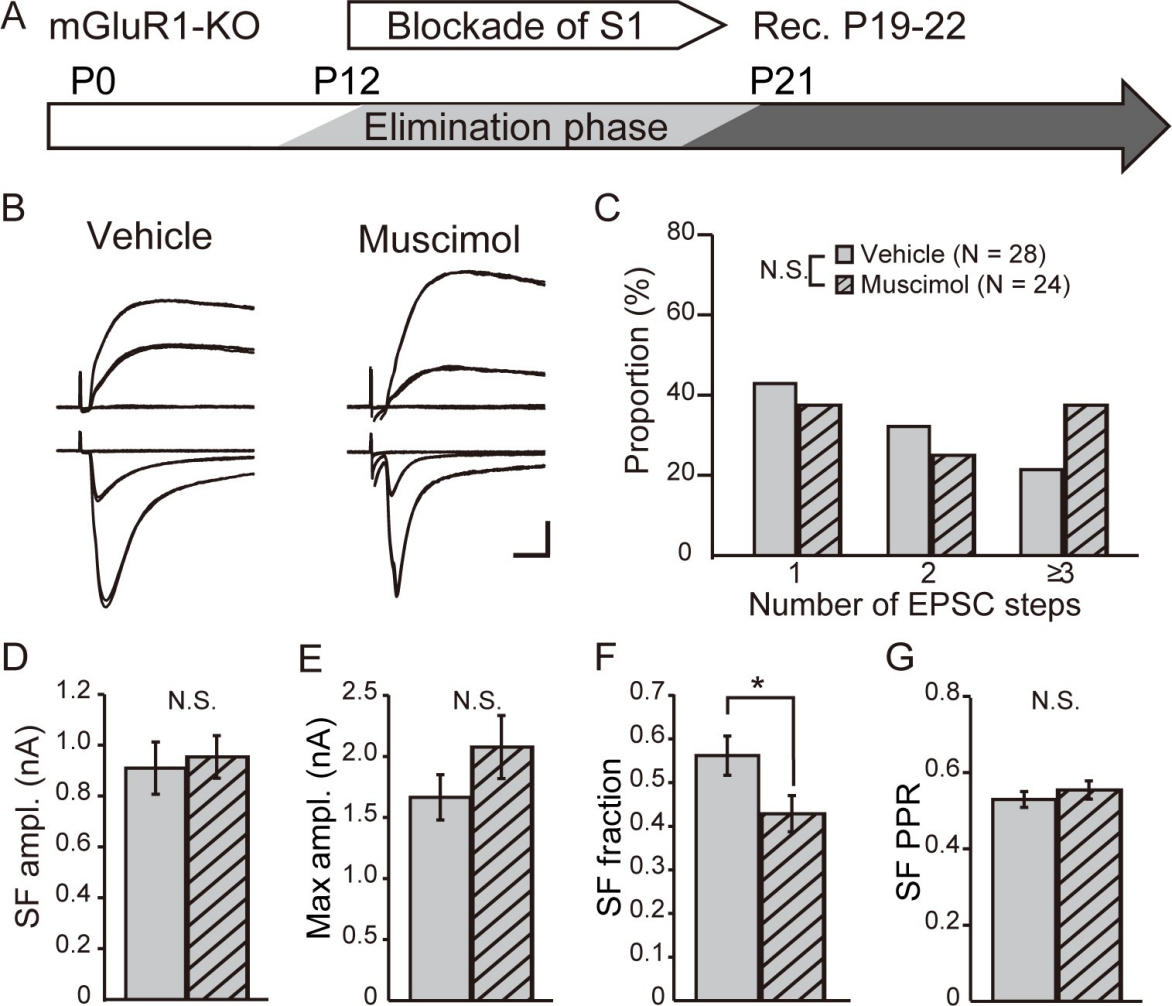
Blockade of cortical activity fails to affect elimination of lemniscal synapses in mGluR1-KO mice. (A) Experimental schedule: muscimol was applied to the cortical S1 area of mGluR1-KO mice from P12. Electrophysiological recordings were obtained from P19 to P22. (B) Representative traces of TC neurons obtained from vehicle-treated (left) or muscimol-treated (right) mGluR1-KO mice. Calibration bars = 0.5 nA and 5 ms. (C) Distribution of step number of lemniscal EPSCs after vehicle (gray) or muscimol (gray-hatched) treatment. N.S., not significant, chi-square test. (D)–(G) Comparisons of the SF AMPAR-mediated EPSC amplitude (D), maximum AMPAR-mediated EPSC amplitude (E), SF fraction (F), and PPR of SF AMPAR-mediated EPSC s (G) between vehicle- (gray) and muscimol-treated (gray-hatched) mGluR1-KO mice. *p < 0.05, Mann–Whitney test. N.S., not significant.

We hypothesized that the activation of mGluR1 by CT input regulates afferent lemniscal synapses. To test this possibility, we perturbed cortical activity by treating mGluR1-KO mice with muscimol during the elimination or maintenance phase. In mGluR1-KO mice that underwent elimination phase application of muscimol to the S1 cortex (Fig 5A), the fraction of cells innervated by multiple lemniscal fibers was similar to that in vehicle-treated mice (vehicle, 53.6%, n = 28 cells vs muscimol, 62.5%, n = 24 cells; P = 0.50, chi-square test; Fig 5B and 5C). The SF AMPAR-mediated EPSC amplitude (vehicle, 0.91 ± 0.10 nA vs muscimol, 0.95 ± 0.08 nA; P = 0.52, Mann–Whitney test) (Fig 5D) and SF PPR (vehicle, 0.53 ± 0.02 vs muscimol, 0.55 ± 0.02; P = 0.45, Mann–Whitney test) (Fig 5G) were not significantly different. The maximum AMPAR-mediated EPSC amplitude (vehicle, 1.67 ± 0.18 nA vs muscimol, 2.08 ± 0.26 nA; P = 0.22 Mann–Whitney test) (Fig 5E) had also no significant difference, but it tended to be large in muscimol-treated mice. Therefore, the SF fraction was lower in muscimol-treated mice (vehicle, 0.56 ± 0.04 vs muscimol, 0.43 ± 0.04; P = 0.02, Mann–Whitney test) (Fig 5F). Overall, elimination phase application of muscimol to mGluR1-KO mice had a small effect on the phenotype of lemniscal EPSCs.

**Fig 6:**
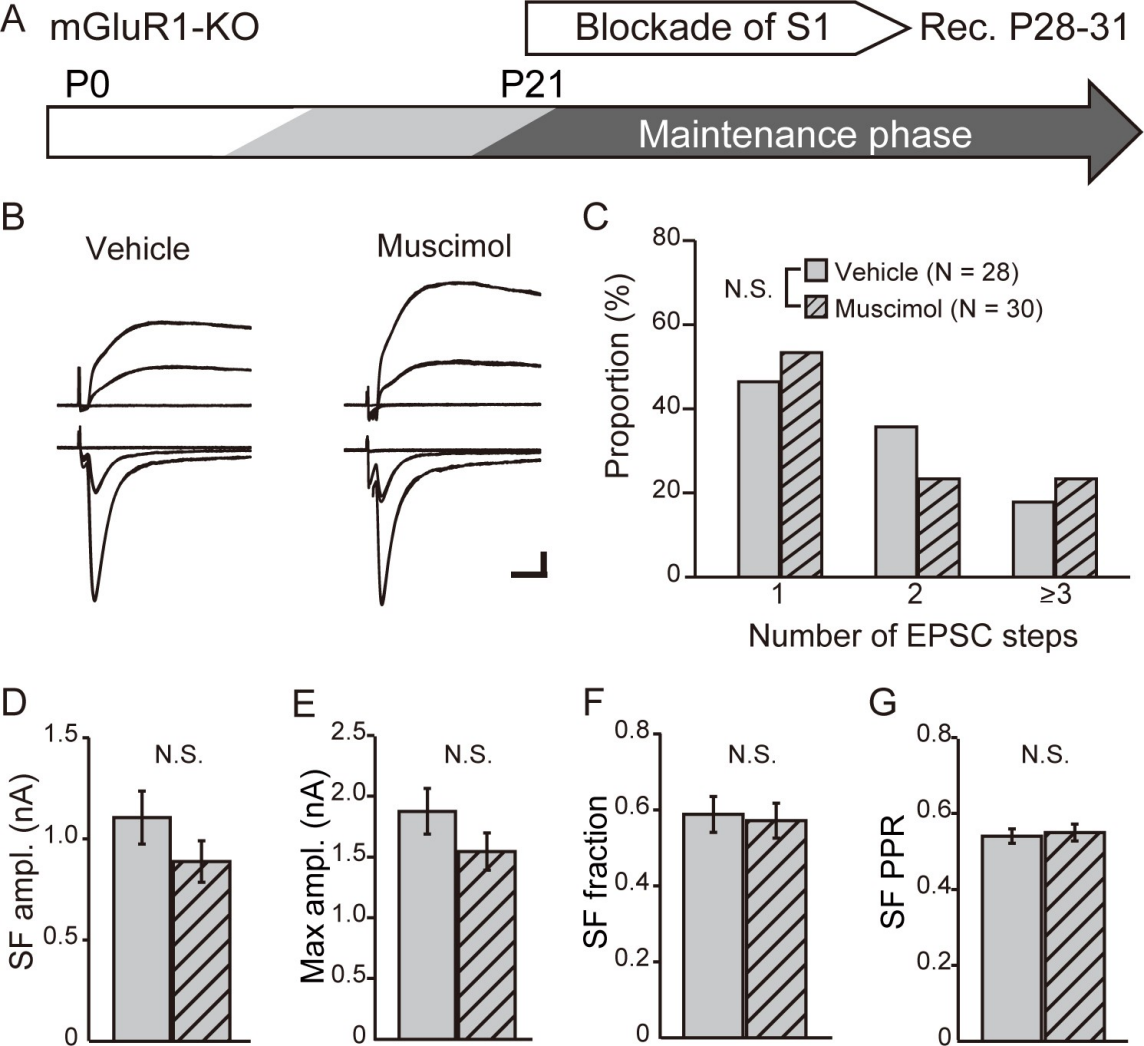
Blockade of cortical activity fails to affect the maintenance of lemniscal synaptic connectivity in mGluR1-KO mice. (A) Experimental schedule: muscimol was applied to the cortical S1 area of mGluR1-KO mice from P21. Electrophysiological recordings were obtained from P28 to P31. (B) Representative traces of TC neurons obtained from vehicle-treated (left) or muscimol-treated (right) mGluR1-KO mice. Calibration bars = 0.5 nA and 5 ms. (C) Distribution of step number of lemniscal EPSCs after treatment with vehicle (gray) or muscimol (gray-hatched). N.S., not significant, chi-square test. (D)–(G) Comparisons of the SF AMPAR-mediated EPSC amplitude (D), maximum AMPAR-mediated EPSC amplitude (E), SF fraction (F), and PPR of SF AMPAR-mediated EPSC s (G) between vehicle- (gray) and muscimol-treated (gray-hatched) mGluR1-KO mice. N.S., not significant, Mann–Whitney test.

Next, to detect the effect of muscimol on the maintenance of lemniscal synaptic connectivity, muscimol was applied to the S1 cortex of mGluR1-KO mice during the maintenance phase (Fig 6A and 6B). After treatment with muscimol for 1 week, the distribution of the step numbers of lemniscal EPSC (Fig 6C) was similar to that after treatment with the vehicle in mGluR1-KO mice (P = 0.91 with chi-square test). Further, the SF AMPAR-mediated EPSC amplitude (vehicle, 1.11 ± 0.13 nA vs muscimol, 0.89 ± 0.10 nA; P = 0.17, Mann–Whitney test) (Fig 6D), maximum AMPAR-mediated EPSC amplitude (vehicle, 1.88 ± 0.19 nA vs muscimol, 1.54 ± 0.15 nA; P = 0.17 with the Mann–Whitney test) (Fig 6E), SF fraction (vehicle, 0.59 ± 0.05 vs muscimol, 0.57 ± 0.05; P = 0.92, Mann–Whitney test) (Fig 6F), and SF PPR (vehicle, 0.54 ± 0.02 vs muscimol, 0.55 ± 0.02; P = 0.99, Mann–Whitney test) (Fig 6G) were not significantly different between the muscimol- and vehicle-treated mice. Together, these data suggest that blocking cortical activity was mainly occluded in mGluR1-KO mice, indicating that the activation of mGluR1 by cortical input regulated afferent lemniscal synaptic connectivity during developmental elimination and maintenance phases.

## Discussion

Here we showed that the activation of mGluR1 plays multiple roles in development and maintenance of afferent synapses in the somatosensory thalamus. mGluR1-KO mice exhibit delayed strengthening, incomplete elimination, and failure to maintain lemniscal synapses. As previously reported, mGluR1 contributes to the elimination and maintenance of synapses [27, 28]. Here we discovered that mGluR1 regulated the strengthening of synapses in VPm. Our data strongly suggest that mGluR1 was most likely activated by CT input because perturbation of cortical activity disrupted strengthening, elimination, and maintenance of synapses in WT mice but had no further impairment in mGluR1-KO mice. Thus, mGluR1 is required for development, refinement, and maintenance of precise neuronal connectivity in the sensory thalamus through the regulation of synaptic strength.

### mGluR1 contributes to the development and refinement of lemniscal synapses

mGluR1 was first identified as a regulator of elimination of synapses in the cerebellum [28]. Subsequent studies suggest that mGluR1 mediates other stages of development, including the maintenance of mature synapses in other regions [27]. In dLGN, mGluR1 maintains mature synaptic connectivity but is not involved in initial formation and strengthening or subsequent elimination of synapses [17]. Most notably, in VPm, our electrophysiological observations during early development (P7–P11) indicated that the absence of mGluR1 was associated with smaller lemniscal EPSC amplitude (Fig 2 and Tables 1 and 2). Therefore, mGluR1 contributes to strengthening of immature lemniscal synapses in the early phase as well as pruning of excess synapses or maintenance of mature synapses in the subsequent phases. Despite the functional similarity of dLGN and VPm as relay centers for sensory information, both of which receive strong sensory afferents and cortical feedbacks as excitatory inputs, mGluR1 differentially contributes to the regulation of synaptic connectivity in VPm and dLGN. The difference in mGluR1 activity may be explained by developmental timing of expression of mGluR1 (Fig 1). Compared with dLGN, where the expression of mGluR1 significantly increased from P21, which is approximately 1 week after eye opening of mice, the expression of mGluR1 in VPm was constant throughout development (Fig 1B). Therefore, compared with retinogeniculate synapses, mGluR1 can regulate the development and maturation of lemniscal synapses in earlier phases. The timings of expression of mGluR1 in the two thalamic nuclei is along with the development of each sensory system, that is, the onset timing of the somatosensory system is earlier than that of the visual system.

The function of group I mGluRs is frequently discussed in the context of synaptic depression [14, 16] to decrease the cell surface expression of AMPARs and remodel spine structures [29–31]. However, type I mGluRs can mediate long-term potentiation in the sensory cortex and hippocampus [32–35]. Such a potentiation mechanism accounts for mGluR1-dependent strengthening of lemniscal synapses early during development.

After elimination of synapses, mGluR1-KO mice gradually exhibited weakening of the existing synapses and recruitment of new synapses (Figs 2 and 3), suggesting that mGluR1 is required to maintain mature synaptic connectivity in VPm. Such group I mGluRs-mediated maintenance of synapses occurs in dLGN [17] as well as in the somatosensory cortex [35, 36] and cerebellum [37]. Transection of the infraorbital nerve that conveys whisker-transduced sensory information to the principle trigeminal sensory nucleus (PrV), the origin of the lemniscal fiber, results in remodeling of lemniscal synaptic connectivity after the elimination phase [22, 38]. This implies activity-dependent maintenance of synaptic connectivity after completion of elimination of synapses, which may exist in VPm despite findings that whisker deprivation at P16 does not induce remodeling of lemniscal synapses [7].

Downstream mechanisms for maintenance of synapses are insufficiently characterized, whereas a molecule, such as MeCP2, stargazin or Fn14 is required for the maintenance of synaptic connectivity in dLGN [10, 12, 39]. Such molecules can act in concert with mGluR1 in VPm to refine and maintain mature neuronal connectivity through weakening, pruning, or both of synapses.

How can mGluR1 play such varied roles during the development of lemniscal synapses? Elimination of excess synapses is independent of previous strengthening because deletion of the genes encoding the GluA3 subunits, GluA4 subunits, or both of AMPARs drastically weakens the lemniscal synaptic strength without affecting the elimination of synapses [40]. Therefore, uncompleted elimination of synapses observed in mGluR1-KO mice would not be caused by delayed strengthening. Conversely, failure to maintain synapses includes weakening of existing synapses; therefore, the maintenance process might share common signal transduction cascades with the strengthening mechanism. Although mGluR1-mediated strengthening of synapses might require mGluR1-dependent LTP mechanisms, downstream pathway components, such as PKCs [32] or Arc [33], are required for elimination of synapses [41, 42]. Thus, the ability of mGluR1 to achieve such an extensive effect on regulation is difficult to solely rationalize through known downstream cascades. Different distributions of mGluR1 within TC neuronal structures during development discussed below may provide an explanation.

### mGluR1 mediates homosynaptic or heterosynaptic regulation of synaptic connectivity

TC neurons receive glutamatergic inputs from sensory afferents and feedback from cortical L6 [43]. Evidence indicates that sensory afferents are drivers, whereas cortical feedback are modulators of thalamic activity during sensory processing [44, 45], and interaction between these two inputs during developmental circuit formation was recently reported [19, 46–48]. For example, surgical or genetic deletion of retinal fiber innervation in dLGN accelerates the timing of cortical fiber innervation [46, 47], whereas retinal ganglion cell axons in mice genetically lacking a cortical structure fail to terminate in dLGN [48]. After synaptic maturation, cortical activity mediates the maintenance of retinogeniculate synaptic connectivity [19]. These findings indicate that the two excitatory inputs can interact by concurrently affecting thalamic neuronal activity through intracellular signal cascades.

Here we show that in VPm, cortical activity regulated the elimination of surplus synapses during development as well as the maintenance of mature synaptic connectivity, as reported in dLGN, because blockade of cortical activity by treatment with muscimol perturbed elimination and maintenance of synapses (Figs 3 and 4). Interestingly, mGluR1-KO mice did not exhibit additional effects of the blockade of cortical activity, suggesting that cortical regulation of elimination and maintenance was mediated by mGluR1 (Figs 5 and 6).

It is not surprising that mGluR1 is involved in heterosynaptic regulation of the elimination of lemniscal synapses because mGluR1-dependent elimination of synapses in the cerebellar climbing fiber-PC synapses is heterosynaptically regulated by the other excitatory input sources to PCs, which are the parallel fibers [28]. Moreover, mGluR1 is necessary and sufficient for visual experience-dependent maintenance of retinogeniculate synapses [17], which requires a precise pattern of cortical activity [19]. Considering that expression of mGluR1 is highly concentrated at the postsynaptic site of CT synapses, cortical feedback is the most adequate source for glutamatergic input for the regulation of elimination and maintenance of lemniscal synapses.

Here we were unable to identify the mechanism underlying the activation of mGluR1 required for strengthening of lemniscal synapses during P7–P11. Ultrastructural analysis revealed that in VP of neonatal mice, mGluR1 is preferentially expressed in the postsynaptic membrane of morphologically identified lemniscal synapses and then changes the localization pattern to the postsynaptic vicinity of CT synapses [26]. Consistent with this report, repetitive stimulation of cortical fibers, which can induce prolonged depolarization in adults, failed to induce mGluR-mediated responses until P8, whereas treatment with an mGluR agonist induced depolarization of the cellular membranes from P0 [49]. These findings indicate that strengthening of lemniscal synapses is more dependent on homosynaptic activation of mGluR1 than on heterosynaptic activation by cortical feedback during early development.

In summary, we demonstrate the multiple roles of mGluR1 in the precise lemniscal synaptic connectivity in VPm. Considering that group I mGluRs are required for the development of synapses and contribute to the pathogenesis of neuropsychiatric diseases [13], our results provide new insights that illuminate the functions of group I mGluRs that orchestrate balanced functional synaptic connectivity during development as well as after maturation.
